# hUMSC-derived exosomes alleviate hypoxic cerebrovascular injury *via* AMPK/NLRP3-mediated pyroptosis suppression and mitochondrial protection

**DOI:** 10.7555/JBR.39.20250189

**Published:** 2026-05-21

**Authors:** Jinteng Liu, Yunlong Pan, Haolin Wu, Qingyun Guo, Xingyue Fang, Yingmei Lu, Qibing Liu

**Affiliations:** 1 Department of Pharmacy & Engineering Research Center of Tropical Medicine Innovation and Transformation, the First Affiliated Hospital & School of Pharmacy of Hainan Medical University, Haikou, Hainan 570102, China; 2 Key Laboratory of Modern Toxicology of Ministry of Education; School of Basic Medical Sciences, Nanjing Medical University, Nanjing, Jiangsu 211166, China; 3 International Research Center on Aging and Tumor, Hainan Medical University, Haikou, Hainan 571199, China; 4 Key Laboratory of Brain Science Research & Transformation in Tropical Environment of Hainan Province, Hainan Medical University, Haikou, Hainan 571199, China

**Keywords:** stroke, hUMSC-Exos, AMPK/NLRP3 signaling pathway, pyroptosis, synergistic combination therapy

## Abstract

As the most prevalent cause of death worldwide, ischemic stroke urgently requires innovative therapeutic strategies. The present study demonstrated the therapeutic potential of human umbilical cord-derived mesenchymal stem cell-derived exosomes (hUMSC-Exos) in ameliorating hypoxia-induced cerebrovascular endothelial dysfunction through modulation of the AMPK/NLRP3 signaling pathway. Bioinformatics analysis of DisGeNET and exosomal cargo databases revealed 283 overlapping cerebral ischemia-related genes, implicating hUMSC-Exos in inflammatory regulation. *In* *vitro* experiments showed that hUMSC-Exos rescued oxygen-glucose deprivation (OGD)-induced endothelial dysfunction in bEnd.3 mouse brain endothelial cells, restoring viability, migration, and mitochondrial integrity. Mechanistically, hUMSC-Exos reversed OGD-induced AMPK inactivation while suppressing NLRP3 inflammasome activation, caspase-1 cleavage, and gasdermin D (GSDMD)-mediated pyroptosis. Molecular docking revealed that DL-3-n-butylphthalide acts as a dual-target ligand for AMPK/NLRP3, synergizing with hUMSC-Exos to enhance endothelial protection. *In* *vivo*, combined therapy in the transient middle cerebral artery occlusion mouse model reduced cerebral infarction and improved neurological outcomes, accompanied by NLRP3/GSDMD downregulation and hippocampal neuron preservation. These findings establish hUMSC-Exos as regulators of AMPK/NLRP3-mediated pyroptosis and propose a translatable combinatorial regimen for ischemic stroke therapy.

## Introduction

Stroke represents the second most prevalent cause of global mortality^[1]^ and a predominant cause of chronic disability worldwide. Despite advances in acute interventions such as thrombolysis and thrombectomy, their narrow therapeutic time windows (< 4.5 h) and incomplete efficacy in mitigating ischemia-reperfusion injury (IRI) underscore the urgent need for novel therapies^[2]^. Ischemic stroke arises from cerebral hypoperfusion, which triggers inflammatory cascades and oxidative stress, leading to irreversible neuronal damage within hours of onset^[3–4]^. Central to this pathology is cerebrovascular endothelial dysfunction, which is crucial for neurovascular coupling, blood-brain barrier (BBB) maintenance, and inflammatory homeostasis^[5–6]^.

Endothelial cells, strategically positioned at the blood-brain interface, govern vascular tone, permeability, and immune responses through dynamic interactions with circulating mediators^[7–11]^. Post-ischemic inflammation exacerbates endothelial injury *via* leukocyte infiltration, cytokine storms (*e*.*g*., TNF-α, IL-6, and IL-1β), and microglial activation, thereby perpetuating oxidative stress and BBB disruption^[12–14]^. Notably, NLRP3 inflammasome-driven pyroptosis—a lytic, pro-inflammatory form of cell death—has emerged as a critical amplifier of IRI. Activated by DAMPs/PAMPs, NLRP3 recruits ASC and pro-caspase-1, leading to the cleavage of gasdermin D (GSDMD) and the induction of pore formation, IL-1β/IL-18 release, and endothelial disintegration^[15–19]^. While NLRP3 inhibitors (*e*.*g*., MCC950) show preclinical promise, their clinical translation remains hampered by systemic toxicity and limited BBB penetration^[20–21]^. Cell-based therapies, particularly mesenchymal stem cells (MSCs), offer neuroprotection by modulating inflammation and promoting angiogenesis. However, risks of pulmonary embolism, tumorigenesis, and poor engraftment limit their utility^[22–23]^. In contrast, MSC-derived exosomes—nanoscale vesicles (30–200 nm) enriched with miRNAs, proteins, and lipids—present a safer alternative. Exosomes recapitulate MSC benefits by suppressing NLRP3, enhancing neurovascular repair, and traversing the BBB without thrombotic or immunogenic risks^[24–26]^. DL-3-n-butylphthalide (NBP), a clinically validated neuroprotectant for ischemic stroke in China, exhibits multimodal protective effects by enhancing cerebral perfusion, attenuating oxidative damage, and suppressing apoptotic pathways. Recent work highlights its NLRP3 inhibitory effects, suggesting synergy with exosomes^[27–29]^. However, the combined efficacy of exosomes and NBP in targeting AMPK/NLRP3 signaling—a nexus of metabolic regulation and pyroptosis—remains unexplored.

The present study aimed to evaluate whether human umbilical cord mesenchymal stem cell-derived exosomes (hUMSC-Exos) synergize with NBP to alleviate cerebral IRI by dual targeting of the AMPK/NLRP3 signaling pathway.

## Materials and methods

### Animals

All experimental procedures were approved by the Institutional Animal Care and Use Committee (IACUC) of Hainan Medical College (Protocol No. HYLL-2022-240). Male C57BL/6J mice (8 weeks old, 20–25 g) were obtained from Guangdong Medical Experimental Animal Center, Guangzhou, China. The mice were kept under a 12-h light/dark cycle with *ad* *libitum* access to food and water, except for food restriction before the anesthesia procedure. All experimental procedures were conducted under anesthesia with 1.5%–2% isoflurane mixed with oxygen and nitrogen. Adult male C57BL/6J mice underwent transient middle cerebral artery occlusion/reperfusion (tMCAO) surgery following standard protocols.

### tMCAO mouse model

Mice were anesthetized with 1.5%–2% isoflurane in a mixture of oxygen and nitrogen and placed on a heating pad to maintain body temperature at 37 (± 1) ℃ throughout the procedure. A silicone-coated nylon filament (0.10–0.15 mm in diameter, adjusted according to arterial size; Cat. #M8502, Changsha Maiyue Biotechnology Co., Ltd., China) was carefully inserted through the right common carotid artery into the internal carotid artery until the tip reached the circle of Willis to occlude the origin of the middle cerebral artery. The occlusion was maintained for 2 h, after which the filament was withdrawn to allow reperfusion for 12 h. Postoperatively, mice were kept warm until full recovery from anesthesia.

After tMCAO induction, neurological deficits were evaluated at 12 h after reperfusion using the modified neurological severity score (mNSS). Animals with comparable impairment were randomly assigned to five experimental groups (*n* = 8 per group) using a computer-generated randomization scheme: (1) sham-operated control, (2) tMCAO, (3) tMCAO + NBP (10 mg/kg), (4) tMCAO + high-dose hUMSC-derived exosomes (H-Exos; 1 × 10^11^ particles per mouse), and (5) tMCAO + H-Exos + NBP. NBP (CSPC NBP Pharmaceutical Co., Ltd., Shijiazhuang, China) was administered intraperitoneally as NBP sodium chloride injection (stock concentration: 25 mg/100 mL), with the dosage converted according to body weight. At 12 h after reperfusion, the corresponding exosomes (for groups 4 and 5) were injected intravenously *via* the tail vein, and NBP (for groups 3 and 5) was administered intraperitoneally.

### Brain tissue collection and protein extraction

Mice were anesthetized with isoflurane and euthanized by cervical dislocation. The brains were rapidly removed and rinsed with ice-cold phosphate-buffered saline (PBS) to eliminate residual blood. The cerebral cortex and hippocampal regions were then carefully dissected on an ice-cold platform. Half of the brain tissue was used for TTC staining, while the remaining half was immediately frozen in liquid nitrogen and stored at −80 ℃ for subsequent sectioning and protein extraction. For protein extraction, frozen brain tissues were thawed on ice and homogenized in RIPA lysis buffer (Cat. #P0013B, Beyotime Biotechnology) supplemented with a protease inhibitor cocktail (Cat. #04693116001, Roche) and a phosphatase inhibitor cocktail (Cat. #04906845001, Roche). The homogenates were incubated on ice for 30 min with intermittent vortexing and then centrifuged at 12000 *g* for 15 min at 4 ℃. The supernatants were collected, and protein concentrations were determined using a BCA protein assay kit (Cat. #23225, Thermo Fisher Scientific). The lysates were either used immediately or stored at −80 ℃ for subsequent Western blot analysis.

### Isolation and characterization of exosomes

**Figure 1:**
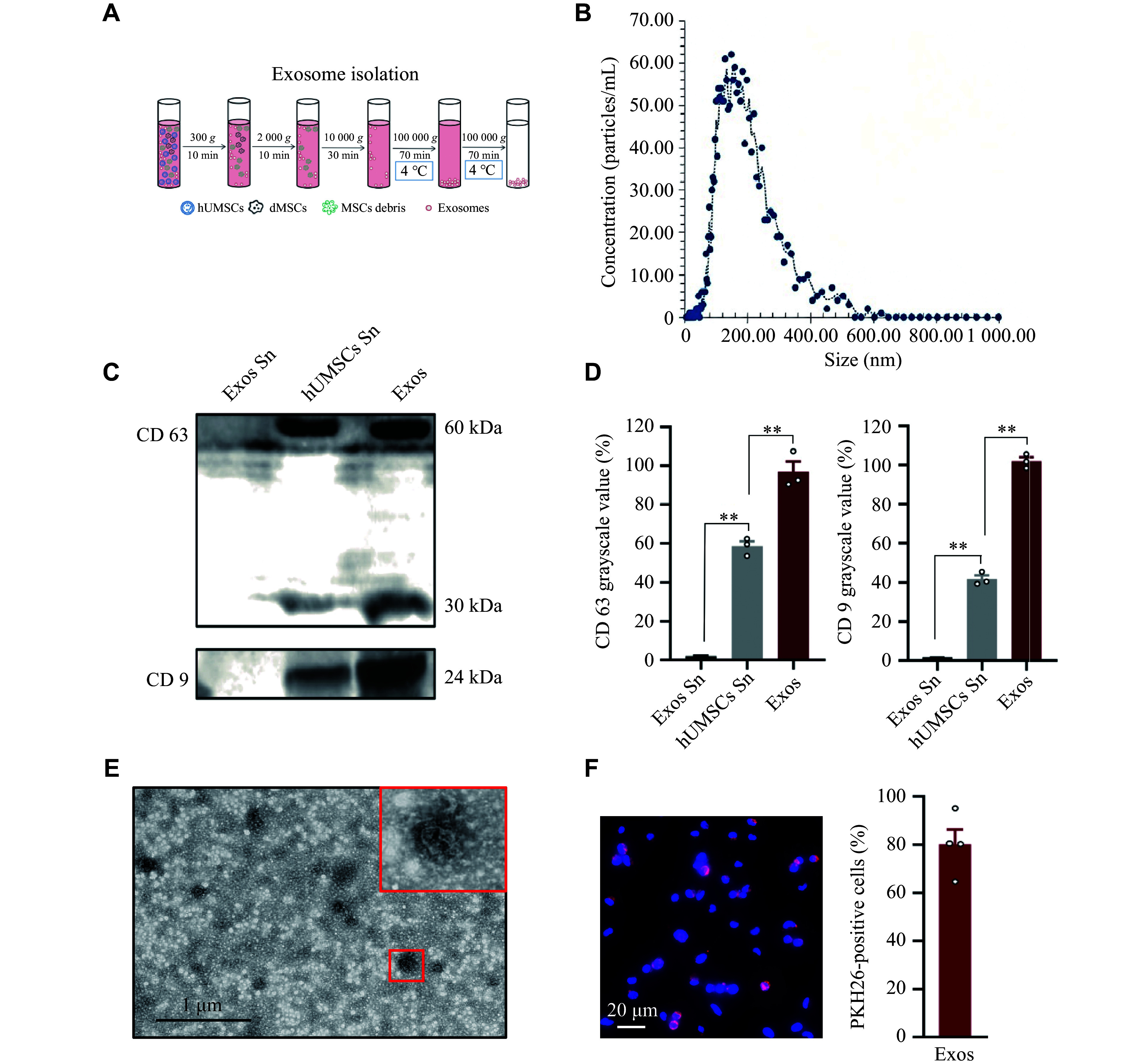
Characterization and functional profiling of exosomes. A: Isolation workflow for human umbilical mesenchymal stem cells (hUMSCs)-derived exosomes using differential ultracentrifugation. B: Nanoparticle tracking analysis of isolated exosomes. C: Western blotting analysis showing CD63 and CD9 expression in the final supernatant after exosome centrifugation (Exos Sn), hUMSCs supernatant (hUMSCs Sn), and exosome precipitates (Exos). D: Quantitative analysis of CD63 and CD9 grayscale values shown in (C). E: Transmission electron microscopy image of isolated exosomes. F: Confocal immunofluorescence images and fluorescence counting showing the uptake of PKH26-labeled exosomes by bEnd.3 cells. Red: exosomes; Blue: DAPI. Scale bar: 20 μm. Data are presented as mean ± standard deviation. ^**^P < 0.01 by one-way ANOVA followed by Tukey's multiple comparisons test.

The MSCs were obtained from the Shanghai Zhong Qiao Xin Zhou Biotechnology Co., Ltd. (Cat. #DF-GMP-ZB09BA, Shanghai, China) with informed consent, under the approved protocol of the Hainan Medical College Ethics Committee (Approval No. HYLL-2023-026). The culture medium was replaced when the MSCs reached 60% confluence, and the conditioned medium was harvested following a 72-h culture period. The conditioned medium was subjected to differential ultracentrifugation (***Fig. 1***) at 4 ℃, and the pellet was resuspended in 2 mL of PBS (Cat. #P1010, Solarbio, Beijing, China) and stored at −80 ℃. The isolated exosomes were systematically characterized through transmission electron microscopy for morphological analysis, nanoparticle tracking analysis for size distribution profiling, and Western blotting (WB) for biomarker validation.

### Oxygen-glucose deprivation (OGD)

*In* *vitro*, bEnd.3 cells were induced with Hanks' Balanced Salt Solution (Cat. #G4204, HBSS, Servicebio, Wuhan, China) to simulate OGD injury, inducing time-dependent cell death. Cells were pretreated with 1 × 10^7^ or 1 × 10^9^ particles/mL exosomes for 1 h, followed by OGD for 6 h. Exosomes remained present during OGD. Cell viability and proliferation capacity were assessed using the Cell Counting Kit-8 (CCK-8) and 5-ethynyl-2′-deoxyuridine (EdU) assays. The expression of target proteins was evaluated by immunofluorescence (IF) staining and immunoblot analysis.

### CCK-8 assay

bEnd.3 cells were cultured to 85% confluence and then diluted to 1 × 10^4^ cells/well. The cells were incubated at 37 ℃ in 5% CO_2_ overnight. After each treatment, 20 μL of CCK-8 working solution (Cat. #C0038, Beyotime Biotechnology, Shanghai, China) was added to each well and incubated for 1 h. The absorbance of each well was measured at 450 nm using a microplate reader (Cat. # Varioskan LUX, Thermo Fisher Scientific, MA, USA), and cell viability was assessed using average optical density.

### Cell scratch assay

bEnd.3 cells were cultured in 35 mm dishes until reaching 95% confluence. A vertical line and three horizontal lines were drawn on the dish cover, and a 200 μL pipette tip was used to quickly scratch the cells vertically in the dish. After washing with PBS, the cells were pretreated with exosomes for 1 h, then subjected to OGD injury. Exosomes remained present during OGD and the subsequent 6-h recovery period. The scratch area was measured at 0 h and 6 h, and the average value was calculated.

### EdU assay

bEnd.3 cells (2.5 × 10^6^) were seeded in a 24-well plate and cultured to 70% confluence for subsequent OGD and exosome protection studies. A final concentration of 10 μmol/L EdU probe (Cat. #C0075S, Beyotime Biotechnology) was added to the plate and incubated for another 2 h in the incubator. Following treatment, the cells were fixed with 4% paraformaldehyde for 15 min and permeabilized with 1% Triton X-100 (Cat. #T8200, Solarbio) for 10 min, followed by incubation with a 1× Hoechst 33342 (Cat. #C1022, Beyotime Biotechnology) solution in the dark for 10 min. Fluorescent microscopy images were acquired, with proliferating cells visualized by orange fluorescence emission.

### Detection of mitochondrial membrane potential (ΔΨm)

Cells were plated uniformly in a 6-well plate at a seeding density of 2 × 10^5^ cells/well. After subsequent OGD and exosome protection studies, the cells were incubated with a 1× JC-1 dye solution (Cat. #C2003S, Beyotime Biotechnology) in a cell culture incubator at 37 ℃, protected from light for 20 min. Then, the cells were washed twice with serum-free medium, and red and green fluorescence images were captured and combined.

### IF staining

bEnd.3 cells were seeded on sterile glass coverslips placed in a 12-well plate and grown until they reached approximately 70%–80% confluence. The cells were then rinsed briefly with warm PBS and fixed with 4% paraformaldehyde (PFA; Cat. #P0099, Beyotime Biotechnology) in PBS for 15 min at room temperature. Following fixation, the cells were washed three times with PBS and permeabilized with 0.1% Triton X-100 in PBS for 10 min. The cells were incubated in a blocking solution (5% bovine serum albumin [BSA] in PBS) for 1 h at room temperature. The cells were then incubated with primary antibodies diluted in the blocking solution overnight at 4 ℃. The following primary antibodies were used: anti-p-AMPK alpha (Thr172) (1∶100; Cat. #AF3423, Affinity Biosciences, Cincinnati, USA), anti-NLRP3 (1∶100; Cat. #ab270449, Abcam), anti-caspase-1 (1∶200; Cat. #A27901, ABclonal, Wuhan, China), anti-GSDMD (1∶200; Cat. #69469T, Cell Signaling Technology, Boston, MA, USA), and anti-IL-1β (1∶100; Cat. #A23484, ABclonal). The samples were then incubated with Alexa Fluor 488 (Cat. #A11008, Thermo Fisher Scientific) and Alexa Fluor 647 (Cat. #A21235, Thermo Fisher Scientific) secondary antibodies (1∶200) for 1 h at room temperature. Finally, the nuclei were counterstained with DAPI-containing mounting medium (Cat. #BL739A, Biosharp, Beijing, China).

Mouse brain tissues were harvested and immediately fixed by immersion in 4% PFA for 24 h at 4 ℃, followed by cryopreservation in a 30% sucrose solution. The tissues were then embedded in optimal cutting temperature (OCT) compound, and frozen sections were cut at a thickness of 5 µm using a cryostat. The sections were mounted onto Superfrost Plus glass slides and allowed to dry. Prior to staining, the slides were rinsed in PBS to remove the OCT compound. For antigen retrieval, the sections were incubated in a pre-warmed citrate-based antigen retrieval buffer, pH 6.0, and heated in a microwave or a water bath for 10–15 min, followed by a cooling period to room temperature. The subsequent steps, including permeabilization (0.3% Triton X-100), blocking (5% BSA), and incubation with primary and fluorescent secondary antibodies (along with DAPI), were performed similarly to the cell staining protocol described above. The following primary antibodies were used: anti-p-AMPK alpha (Thr172) (1∶100; Cat. #AF3423, Affinity Biosciences) and anti-NLRP3 (1∶50; Cat. #ab270449, Abcam). The images were taken using a fluorescence microscope (Cat. #BX35, Olympus, Tokyo, Japan). Fluorescence intensity was quantified using ImageJ software (V 1.54, NIH, USA). Images were converted to 8-bit grayscale format before analysis. Regions of interest were selected, and the integrated density after background subtraction was measured and used for statistical analysis.

### WB analysis

Cells were lysed using RIPA buffer (Cat. #P0013B, Beyotime Biotechnology), and protein concentration was quantified using a BCA assay kit (Cat. #23225, Thermo Fisher Scientific). After SDS-PAGE separation and electroblotting, PVDF membranes (Cat. #ISEQ00010, Immobilon, Darmstadt, Germany) were blocked with 5% skimmed milk powder dissolved in TBST (Cat. #G0001, Servicebio) and then incubated with the primary antibodies at 4 ℃ overnight. The following primary antibodies were used: anti-AMPK (1∶2000; Cat. #ab32047, Abcam), anti-p-AMPK (1∶2000; Cat. #ab133448, Abcam), anti-NLRP3 (1∶1000; Cat. #ab270449, Abcam), anti-caspase-1 (1∶2000; Cat. #A27901, ABclonal), anti-GSDMD (1∶2000; Cat. #69469T, Cell Signaling Technology), and anti-IL-1β (1∶1000; Cat. #A23484, ABclonal). The PVDF membranes were then incubated with an HRP-labeled goat anti-rabbit IgG (H+L) antibody (1∶1000) (Cat. #A0208, Beyotime Biotechnology) for 2 h at room temperature. Chemiluminescence signals were excited with BeyoECL Plus (Cat. #P0018S, Beyotime Biotechnology) and captured using an imaging system (Cat. #978978, Thermo Fisher Scientific).

### shRNA-mediated *Prkaa1* (encoding the AMPKα1 subunit) knockdown

Small hairpin RNA (shRNA) targeting *Prkaa1* (sh*Prkaa1*) was purchased from Banma Biotechnology Co., Ltd. (Cat. #BM20241230002-3, Changsha, China). The target sequence for *Prkaa1* shRNA was 5*'*-TCAGTACACCATCTGATATTT-3*'*, and the scrambled shRNA sequence was 5*'*-GCATTATTCGCTTTACAACAT-3*'*. Cells were transfected with shRNA plasmids using Lipofectamine 3000 (Thermo Fisher Scientific) according to the manufacturer's instructions. Knockdown efficiency was verified by WB analysis.

### Molecular docking analysis

Ischemia-related target genes were obtained from the DisGeNET database (DisGeNET v4.0; accessed August 2023) using the search term "cerebral ischemia". Candidate genes were prioritized based on gene-disease association (GDA) scores, and two key targets, AMPK catalytic subunit alpha1 (PRKAA1) and NLRP3, were selected for further analysis because of their high GDA scores (> 0.3) and well-established involvement in the pathophysiology of cerebral ischemia. The three-dimensional (3D) structures of AMPK and NLRP3 were retrieved from the RCSB Protein Data Bank. The 2D and 3D molecular structures of NBP (CID 108128) were downloaded from the PubChem database (accessed June 2024). Molecular docking between NBP and the target proteins was performed using AutoDock 4.0. The resulting binding conformations were subsequently visualized and analyzed using PyMOL (v2.5.7).

### 2,3,5-Triphenyltetrazolium chloride (TTC) staining

After reperfusion for 24 h, C57BL/6J mice were euthanized. The brains were cooled at −20 ℃ for 25 min to slightly harden the tissue. Five 2-mm coronal sections were made from the olfactory bulb to the cerebellum, and then stained with TTC (Cat. #C0651, Beyotime Biotechnology). Photographs of the stained brain slices were captured using a digital camera. Image analysis software (SigmaScan Pro 5) was used to measure the infarct area of each brain in a blind manner. The total volumes of both the contralateral and ipsilateral hemispheres, as well as the volumes of the striatum and cortex in both hemispheres, were measured. The infarct percentage was calculated relative to the contralateral structures to avoid erroneous measurements due to edema.

### Hematoxylin-eosin (H&E) staining

Tissue sections were deparaffinized in xylene (10 min × 2), rehydrated through a graded ethanol series (100%, 95%, 80%, and 70%; 3 min each), and rinsed in distilled water. Nuclear staining was performed using an H&E stain kit (Cat. #C0105S, Beyotime Biotechnology). Slides were air-dried at 25 ℃ for 24 h before microscopic examination.

### Statistical analysis

The data are presented as mean ± standard deviation. All statistical analyses were performed using GraphPad Prism 9.1 software (GraphPad Software, San Diego, CA, USA). Biological replicates (*n* ≥ 3) were performed for each experiment. Intergroup comparisons were conducted using two-tailed unpaired Student's *t*-tests or one-way ANOVA followed by Tukey's post hoc test for more than two groups. A *P*-value < 0.05 was considered statistically significant.

## Results

### Characteristic analysis of hUMSC-Exos

To systematically explore the therapeutic potential of hUMSC-Exos in cerebral ischemia, we first demonstrated their molecular and functional relevance. We mined the DisGeNET database and identified 1313 cerebral ischemia-associated genes, of which 283 overlapped with the known exosome cargo components, as revealed by Venn diagram analysis (***Supplementary Fig. 1***). Gene Ontology (GO) enrichment analysis was performed across three domains: biological processes (BP), molecular functions (MF), and cellular components (CC), highlighting significant associations between cerebral ischemia pathogenesis and inflammatory response pathways (***Supplementary Fig. 2***).

Exosomes function as essential intercellular signaling mediators through their cargo of bioactive molecules, including proteins, lipids, DNA, and RNA. Emerging evidence suggests that the therapeutic effects of MSCs are largely mediated by the paracrine secretion of these vesicles. In the present study, exosomes were efficiently isolated from umbilical cord-derived MSCs by differential centrifugation (***Fig. 1A*** and ***1B***). WB analysis demonstrated robust expression of exosome biomarkers CD9 and CD63 in both post-centrifugation PBS supernatants and purified exosome resuspension solutions, whereas these markers were not detected in unconditioned MSC culture supernatants (***Fig. 1C*** and ***1D***), indicating successful exosome enrichment. Nanoparticle tracking analysis (***Fig. 1B***) and transmission electron microscopy (***Fig. 1E***) further characterized the isolated exosomes, revealing their classic spherical morphology with a size distribution of 30–150 nm (mean particle size: 149 nm), consistent with published exosome profiles. High-resolution fluorescence microscopy revealed efficient internalization of PKH26-labeled exosomes by bEnd.3 cells after 6 h of co-culture (***Fig. 1F***), laying the foundation for subsequent functional studies.

### hUMSC-Exos alleviated OGD-induced deficits in bEnd.3 cell viability, migration, and mitochondrial integrity

**Figure 2:**
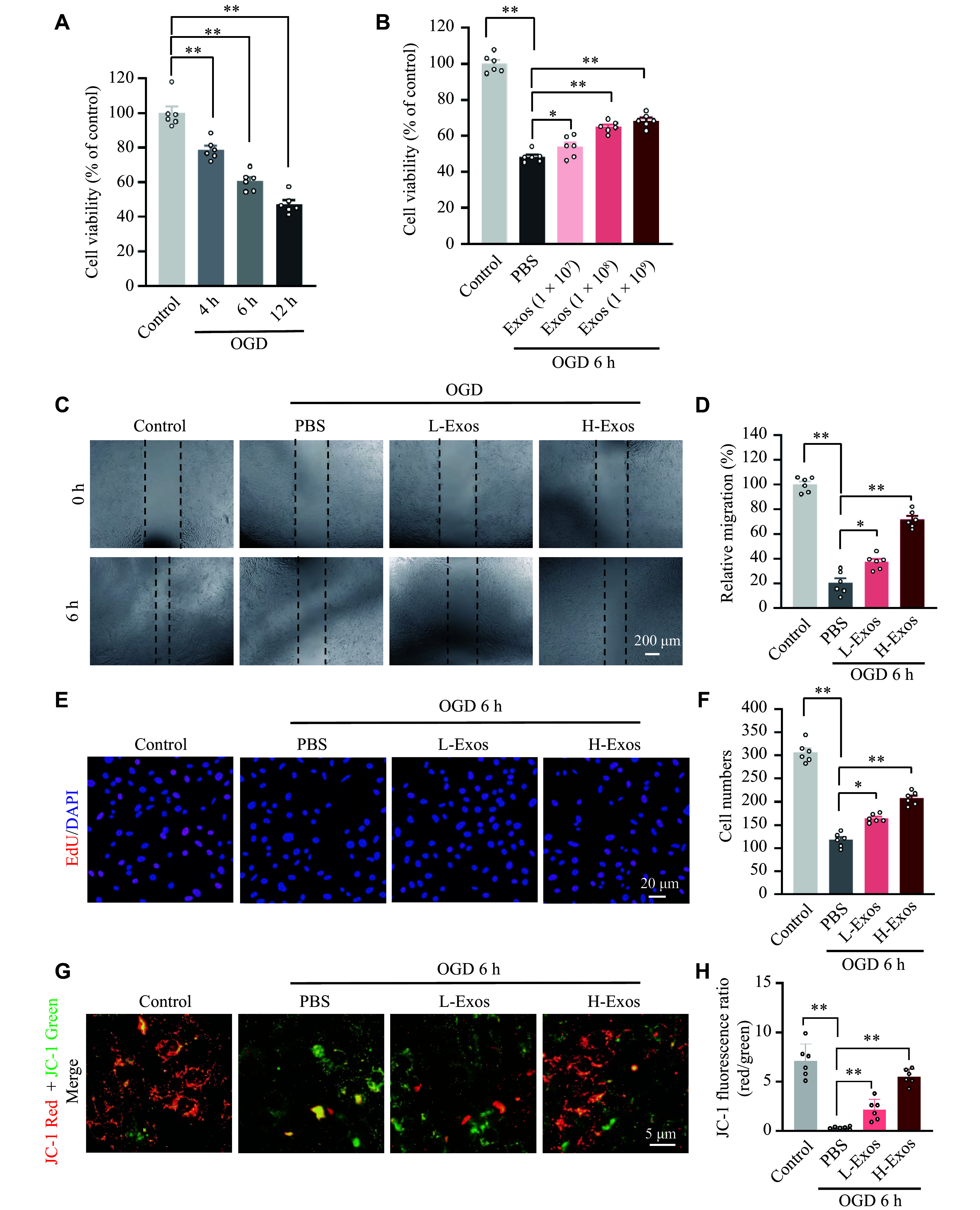
Exosomes attenuated oxygen-glucose deprivation (OGD)-induced impairments in cerebral microvascular endothelial cell function. A: Viability of bEnd.3 cells after 4, 6 and 12 h of OGD. B: Viability of bEnd.3 cells at different concentrations of exosomes after 6 h of OGD. C: Migration of bEnd.3 cells at low-dose (1 × 10^7^ particles/mL, L-Exos) or high-dose (1 × 10^9^ particles/mL, H-Exos) of exosomes after 6 h of OGD. D: Quantification of migration rates shown in (C). E: EdU incorporation assay showing proliferative activity of bEnd.3 cells at L-Exos or H-Exos after 6 h of OGD. F: Quantification of EdU-positive cells shown in (E). G: Representative JC-1 staining image of bEnd.3 cells at L-Exos or H-Exos after 6 h of OGD. H: Quantification of JC-1 fluorescence intensity shown in (G). Data are presented as mean ± standard deviation. ^*^P < 0.05 and ^**^P < 0.01 by two-way ANOVA followed by Tukey's multiple comparisons test.

Based on the evidence of exosome internalization, we investigated whether hUMSC-Exos protect the cerebrovascular endothelium against OGD, a key pathological feature of cerebral ischemia. The CCK-8 assay demonstrated a time-dependent cytotoxic effect of OGD on bEnd.3 cells. Compared with the normoxic control group, the OGD (4–12 h) groups exhibited a significant reduction in the viability of bEnd.3 cells (***Fig. 2A***). Exosome treatment significantly improved cellular viability under OGD (6 h) conditions (***Fig. 2B***). Notably, inverted microscopy observations revealed distinct morphological alterations across groups: bEnd.3 cells in normoxic controls retained their characteristic cobblestone-like morphology and formed a confluent monolayer, whereas OGD-treated cells displayed enlarged surface areas, membrane rupture, and disrupted network architecture after 6 h of OGD. Moreover, scratch assays at 6 h post-injury demonstrated that OGD treatment significantly impaired the migration capacity of bEnd.3 cells (*P* < 0.01 *vs*. control; ***Fig. 2C*** and ***2D***), while exosome pretreatment significantly restored the migration capacity of OGD-treated cells to near-normal levels. Furthermore, EdU incorporation assays revealed that exosomes reversed OGD-induced suppression of cellular proliferation (***Fig. 2E*** and ***2F***). Concurrently, JC-1 fluorescence analysis demonstrated that exosome treatment significantly restored the mitochondrial membrane potential ($\Delta \Psi {\mathrm{m}} $) in OGD-treated cells (***Fig. 2G*** and ***2H***), implicating mitochondrial protection as a potential mechanism.

### hUMSC-Exos mitigated OGD-induced injury *via* AMPK-dependent suppression of NLRP3 inflammasome activation

**Figure 3:**
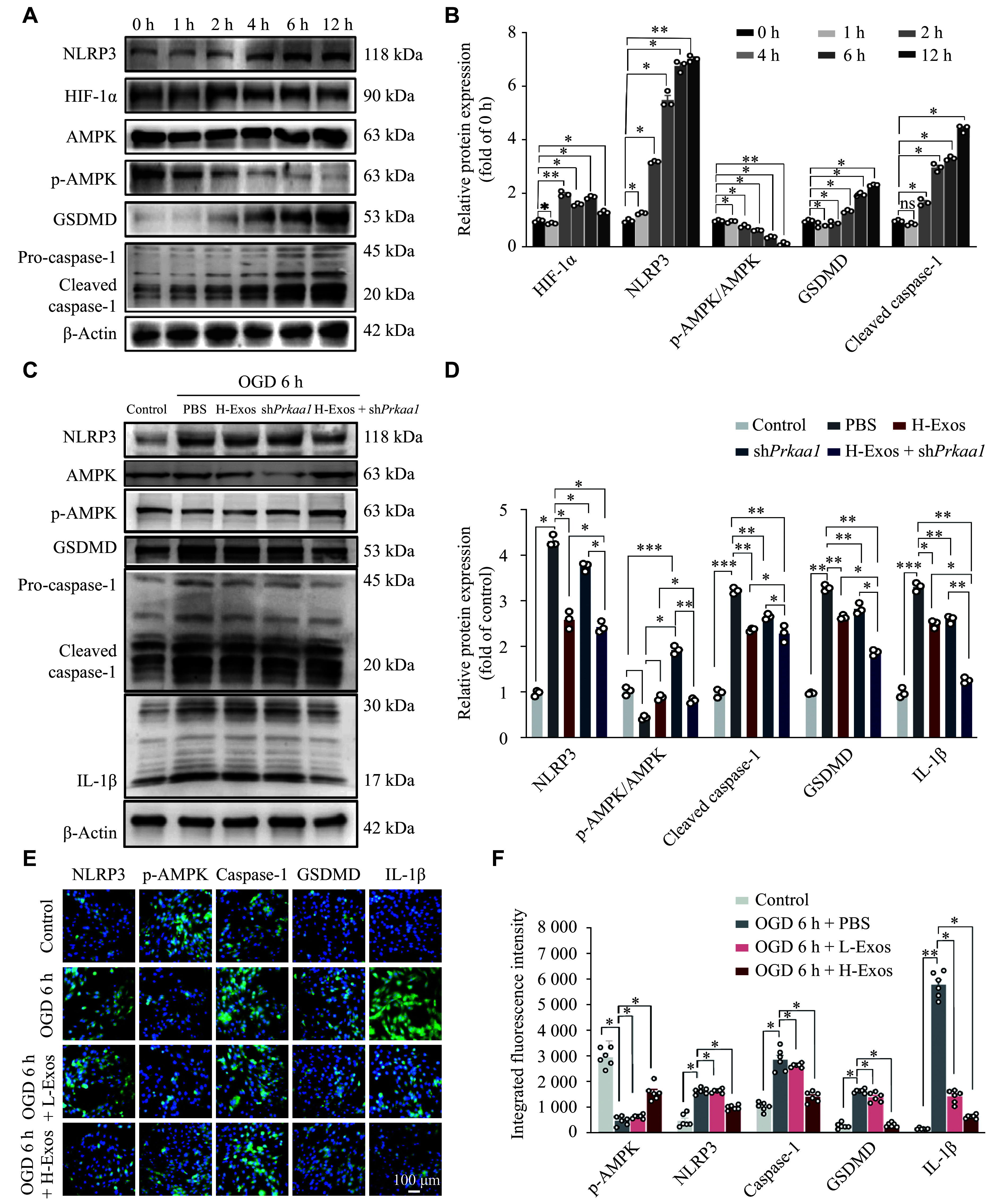
Exosome intervention modulated AMPK and NLRP3 pathway activation during oxygen-glucose deprivation (OGD) injury. A: Representative bands of NLRP3, HIF-1α, AMPK, phosphorylated AMPK (p-AMPK), GSDMD-N, and cleaved caspase-1 expression in time-dependent OGD-exposed bEnd.3 cells by Western blotting analysis. B: Semi-quantitative analyses of protein levels shown in (A), using β-actin as the reference (*n* = 3). C: Representative bands of NLRP3, AMPK, p-AMPK, cleaved IL-1β, cleaved caspase-1, and GSDMD protein expression in bEnd.3 cells after 6 h of OGD with high-dose Exos (H-Exos, 1 × 10^9^ particles/mL), sh*Prkaa1*, and H-Exos + sh*Prkaa1*. D: Semi-quantitative analyses of protein levels shown in (C), using β-actin as the reference (*n* = 3). E: Immunofluorescence staining of NLRP3 (green), p-AMPK (green), caspase-1 (green), GSDMD (green), IL-1β (green), and DAPI (blue) in bEnd.3 cells after 6 h of OGD with low-dose (L-Exos, 1 × 10^7^ particles/mL) or high-dose (H-Exos, 1 × 10^9^ particles/mL) exosomes. F: Quantitative analysis of integrated fluorescence intensity of p-AMPK, NLRP3, caspase-1, GSDMD, and IL-1β in different groups using Image J software (E; *n* = 6). Data are presented as mean ± standard deviation. ^*^P < 0.05, ^**^P < 0.01, and ^***^P < 0.001 by two-way ANOVA followed by Tukey's multiple comparisons test.

Given the observed mitochondrial protection and the bioinformatic link to inflammation, we hypothesized that hUMSC-Exos could modulate AMPK/NLRP3 signaling, a pathway integrating metabolic stress and inflammasome activation. WB analysis revealed that 6 h of OGD suppressed AMPK phosphorylation (indicating inactivation) but activated NLRP3 inflammasome components (***Fig. 3A*** and ***3B***).

To further investigate the AMPK-dependent suppression of NLRP3 inflammasome activation, we performed *Prkaa1* knockdown experiments using shRNA (sh*Prkaa1*). Interestingly, our results revealed that AMPK silencing increased its phosphorylation level under OGD conditions, which may be related to a feedback regulatory mechanism, thereby significantly reducing the inflammatory response induced by OGD, similar to the anti-inflammatory effects observed with exosome therapy. Notably, the combination of H-Exos and *Prkaa1* knockdown exhibited significant synergistic effects, further attenuating inflammation (***Fig. 3C*** and ***3D***). Moreover, IF staining revealed that H-Exos restored AMPK phosphorylation in OGD-injured cells (***Fig. 3E*** and ***3F***), concurrently suppressing NLRP3-driven pyroptosis. This AMPK/NLRP3 axis modulation provides a molecular basis for the observed functional recovery in cellular viability, migration, and mitochondrial integrity.

### hUMSC-Exos combined with NBP synergistically ameliorated OGD-induced bEnd.3 cell injury through coordinated AMPK/NLRP3 pathway modulation

**Figure 4:**
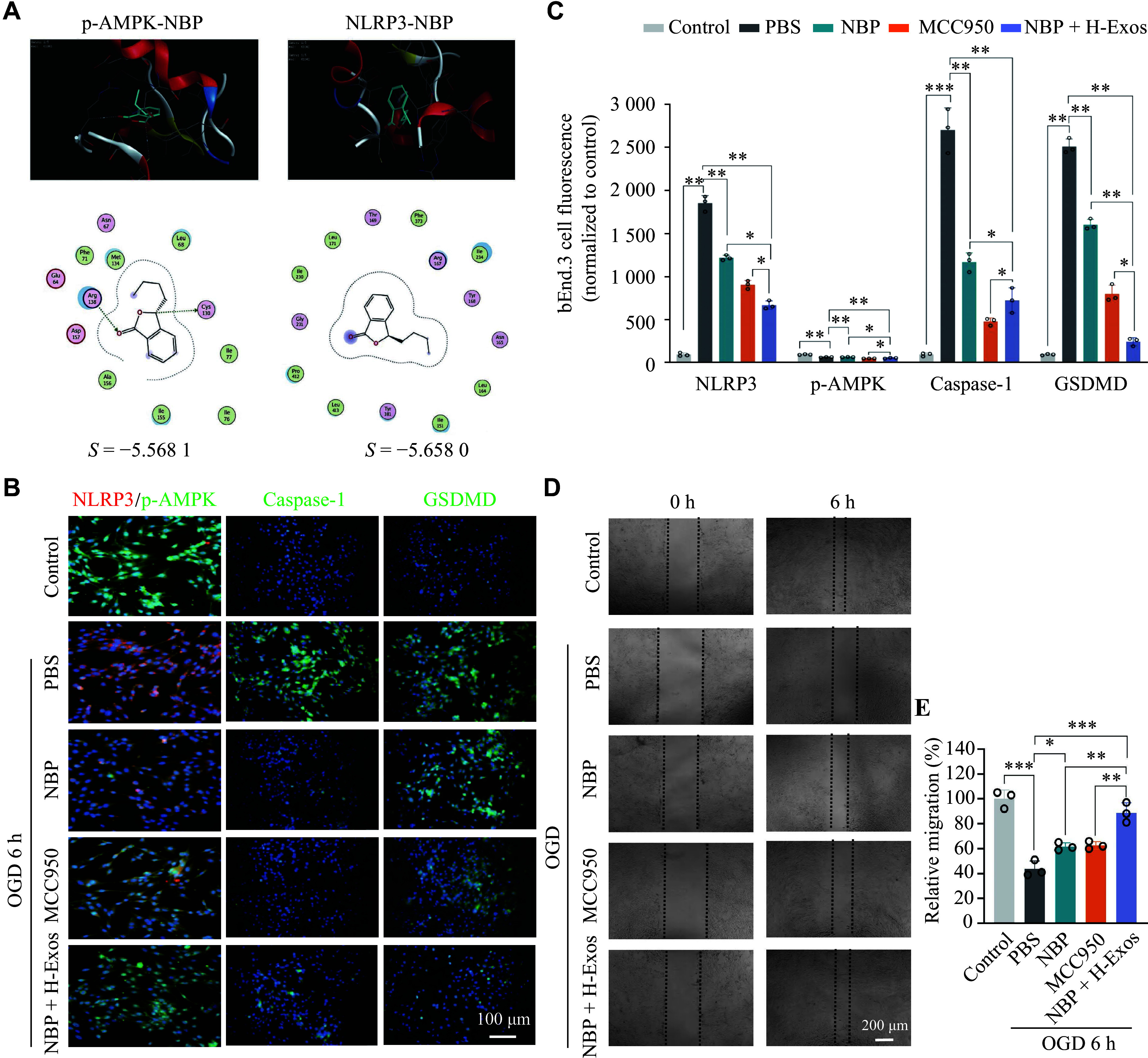
Combinatorial therapy with exosomes and DL-3-n-butylphthalide (NBP) modulated cellular responses to oxygen-glucose deprivation (OGD) injury through the AMPK/NLRP3 pathway. A: Molecular docking analysis of NBP with phosphorylated AMPK (p-AMPK) or NLRP3. B: Representative immunofluorescence staining micrographs of mouse brain microvascular endothelial cells (bEnd.3 cells) for NLRP3 (red), p-AMPK (green), cleaved caspase-1 or GSDMD (green), and nuclei (DAPI, blue). C: Quantification of immunofluorescence intensity shown in (B) (*n* = 3). D: Representative images of a cell migration assay performed with bEnd.3 cells. E: Quantification of the relative migration area shown in (D; *n* = 3). Low-Exos (L-Exos, 1 × 10^7^ particles/mL), high-Exos (H-Exos, 1 × 10^9^ particles/mL). Data are presented as mean ± standard deviation. ^*^P < 0.05, ^**^P < 0.01, and ^***^P < 0.001 by two-way ANOVA followed by Tukey's multiple comparisons test.

Previous studies have demonstrated the therapeutic potential of NBP in cerebral ischemia, where endothelial inflammatory responses play pivotal roles in immunomodulation. We conducted a molecular docking analysis and identified robust binding affinities of NBP with key pathway components: phosphorylated AMPK (p-AMPK; binding energy: −5.5681 kcal/mol) and NLRP3 protein (−5.6580 kcal/mol; ***Fig. 4A***), suggesting its potential to directly interfere with inflammasome activation. Additionally, IF staining revealed that NBP treatment (20 μmol/L) enhanced AMPK phosphorylation in cerebral endothelial cells subjected to 6 h of OGD, thereby inhibiting the NLRP3 inflammasome assembly and restoring endothelial migratory capacity. Notably, combined hUMSC-Exo (1 × 10^9^ particles/mL) and NBP therapy exhibited synergistic effects, surpassing monotherapy outcomes in reversing OGD-induced endothelial injury. Meanwhile, we used MCC950 (Cat. #HY-12815, MedChemExpress, Princeton, NJ, USA) to inhibit NLRP3 expression, further confirming the function of combined hUMSC-Exo and NBP therapy. This combinatorial approach not only amplified AMPK activation but also more effectively suppressed NLRP3-driven pyroptotic signaling, as evidenced by the near-complete restoration of cellular viability and functional integrity (***Fig. 4B***–***4E***). These findings indicate that both hUMSC-Exos and NBP convergently target the NLRP3 inflammasome pathway to mitigate pyroptosis.

### hUMSC-Exos and NBP co-treatment reduced cerebral infarction and suppressed pyroptosis in tMCAO mice *via* AMPK/NLRP3 signaling

**Figure 5:**
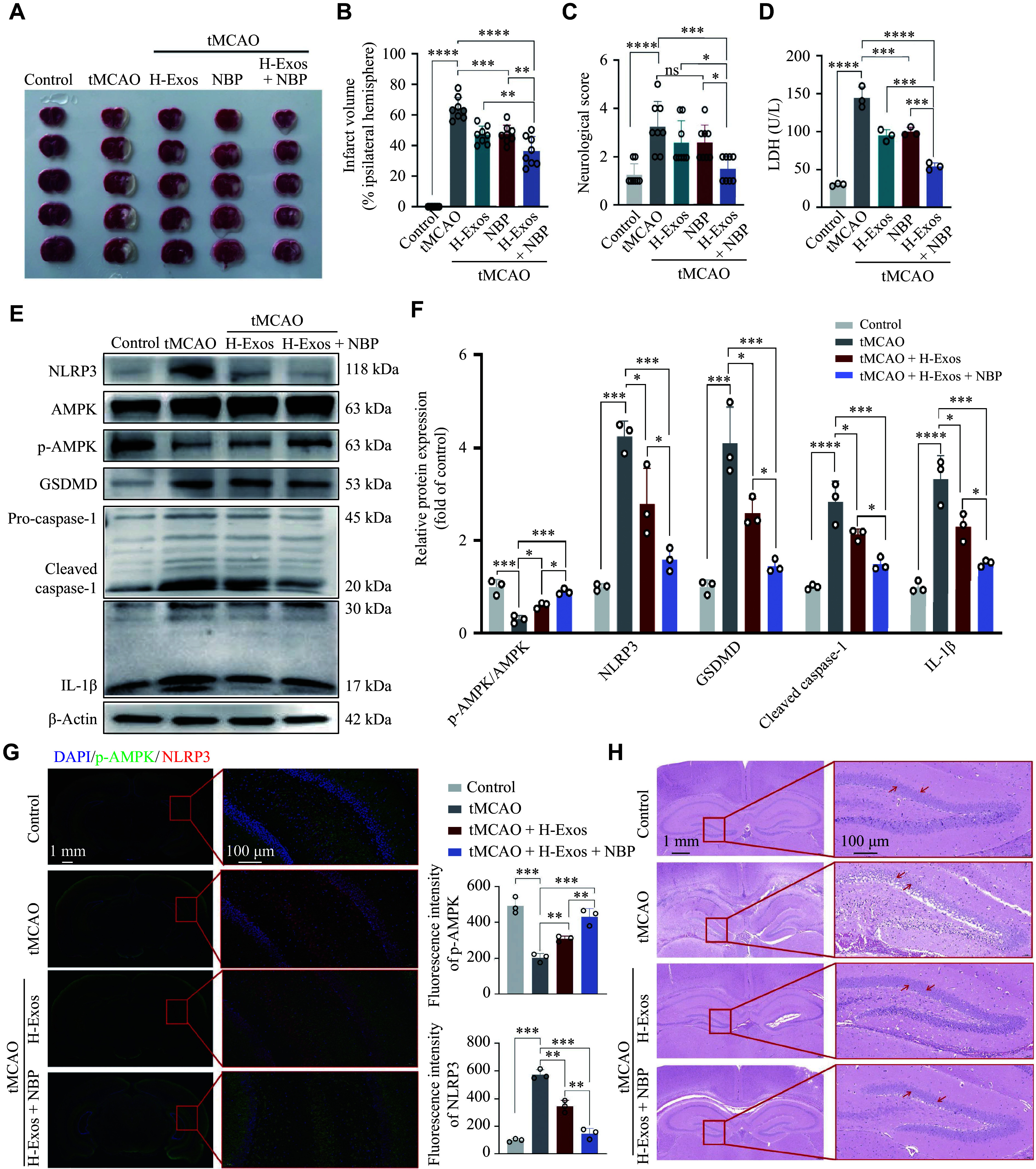
Exosomes attenuated cerebral ischemic injury by modulating the NLRP3 signaling pathway and preserving neurovascular integrity. Mice underwent transient middle cerebral artery occlusion followed by reperfusion (tMCAO) and received the indicated treatments. A: Representative images of 2,3,5-triphenyltetrazolium chloride (TTC)-stained brain sections from C57BL/6 mice subjected to tMCAO. B: Quantification of cerebral infarct volume from TTC staining results shown in (A). C: Neurological deficit assessment in tMCAO mice was evaluated using the modified neurological severity score (mNSS). D: LDH release assay from mouse plasma. E: NLRP3, AMPK, phosphorylated AMPK (p-AMPK), cleaved caspase-1, GSDMD, and IL-1β protein levels were examined by Western blotting. β-Actin was used as the loading control. F: Quantification of protein levels shown in (E) (*n* = 3). G: Immunofluorescence staining and semi-quantitative analysis of p-AMPK (green) and NLRP3 (red) in the cerebral cortex of tMCAO mice (*n* = 3). H: Representative images of hippocampal morphology from brain sections stained with H&E in tMCAO mice. Data are presented as mean ± standard deviation. ^*^P < 0.05, ^**^P < 0.01, and ^***^P < 0.001 by two-way ANOVA followed by Tukey's multiple comparisons test.

Given the *in* *vitro* evidence of synergistic protection, we evaluated the therapeutic efficacy of hUMSC-Exos combined with NBP in the tMCAO mouse model. TTC staining at 24 h post-reperfusion revealed that high-dose hUMSC-Exo monotherapy (intravenous administration, 1 × 10^11^ particles) decreased infarct volume to 48.11% ± 5.88%, NBP monotherapy (intraperitoneal injection, 10 mg/kg) decreased infarct volume to 47.85% ± 5.77%, and high-dose hUMSC-Exo + NBP therapy reduced infarct volume to 36.36% ± 9.54%, all of which were significantly smaller than that of the model group (64.25% ± 7.30%; *P* < 0.01; ***Fig. 5A*** and ***5B***). The neurological score analysis showed that the mNSSs were significantly decreased in the hUMSC-Exo (2.59 ± 0.74), NBP (2.63 ± 0.92), and hUMSC-Exo + NBP (1.50 ± 0.53) groups, compared with the model group at 12 h post-reperfusion (3.25 ± 1.04; *P* < 0.01; ***Fig. 5C***). Further validation in mouse plasma demonstrated that stroke intervention was accompanied by increased lactate dehydrogenase (LDH) release ([144.46 ± 14.30] U/L). Combination therapy reduced LDH levels to (54.09 ± 4.71) U/L (*P* < 0.01 *vs*. the hUMSC-Exo monotherapy group), indicating enhanced membrane integrity (***Fig. 5D***). To elucidate the AMPK/NLRP3-mediated pyroptosis mechanism, we quantified pathway biomarkers by WB analysis. Twenty-four hours post-ischemia-reperfusion, the model group exhibited upregulated levels of NLRP3, cleaved caspase-1, and GSDMD, compared with the sham-operated group (*P* < 0.01). High-dose hUMSC-Exos monotherapy significantly upregulated AMPK phosphorylation and downregulated NLRP3 pathway proteins (*i*.*e*., NLRP3, caspase-1, and GSDMD), while high-dose hUMSC-Exo + NBP co-treatment demonstrated superior efficacy (***Fig. 5E*** and ***5F***). Additionally, IF staining in the mouse hippocampal region demonstrated that high-dose hUMSC-Exo combined with NBP significantly increased p-AMPK expression but decreased NLRP3 protein expression (***Fig. 5G***). H&E staining revealed distinct histopathological features. The control hippocampi demonstrated intact neuronal architecture with well-defined cellular margins and eosinophilic cytoplasm. Conversely, tMCAO specimens showed marked pyramidal cell layer attenuation, sparse arrangement, pale nuclei, blurred boundaries, and nuclear shrinkage in the hippocampal CA1 region. In contrast, the high-dose hUMSC-Exo group displayed more preserved neuronal morphology with relatively intact cellular boundaries and denser cell arrangement. Notably, the high-dose hUMSC-Exo combined with the NBP group exhibited the most pronounced protective effect, characterized by a more complete neuronal structure, clearer nuclear morphology, and more tightly arranged cells than either the model group or the single high-dose hUMSC-Exo group (***Fig. 5H***). Collectively, these findings indicate that stem cell-derived exosomes may potentiate the neurorestorative effects of NBP through dual mechanisms.

## Discussion

In the present study, we elucidated the neuroprotective potential of hUMSC-Exos combined with NBP in mitigating endothelial pyroptosis and cerebral IRI. Our findings indicate that early OGD downregulates AMPK phosphorylation, thereby activating the NLRP3-caspase-1-GSDMD pyroptotic pathway. Co-treatment with hUMSC-Exos and NBP synergistically suppressed these pathways in both OGD-injured endothelial cells and the tMCAO mouse model, highlighting their dual therapeutic potential in modulating inflammatory cell death and preserving neurovascular integrity.

Post-ischemic inflammation is a well-established driver of secondary brain injury, exacerbating neuronal apoptosis and long-term functional deficits^[30]^. While thrombolysis and thrombectomy remain frontline interventions, their inability to address delayed inflammatory cascades underscores the need for adjunct therapies. In this context, our combinatorial approach, targeting AMPK/NLRP3 signaling, significantly reduced cerebral infarct volume, edema, and neurological deficits in tMCAO mice, while attenuating NLRP3 inflammasome activation and IL-1β release. These results align with emerging evidence that the inhibition of pyroptosis through suppression of the NLRP3-GSDMD axis represents a viable strategy to improve stroke outcomes^[31–32]^. Although the present study demonstrated that hUMSC-Exos conferred neurovascular protection *via* AMPK/NLRP3-mediated modulation of pyroptosis and mitochondrial dysfunction, the long-term sustainability of these therapeutic effects requires further evaluation. Furthermore, future studies should investigate potential off-target effects arising from AMPK or NLRP3 modulation, particularly in non-targeted brain cell populations and peripheral tissues, to ensure the specificity and safety of this approach.

hUMSC-Exos (30–150 nm), which are natural nanoscale vesicles, facilitate intercellular communication through the transfer of bioactive molecules, including microRNAs, functional proteins, and lipid mediators. Their clinical potential is underscored by their roles in cancer diagnostics^[33–34]^ and cardiovascular repair^[35]^, yet challenges persist in scalable production and therapeutic efficacy. Although hUMSC-Exos alone demonstrate partial neuroprotection, their limited yield and transient bioactivity necessitate combinatorial strategies. Notably, NBP, a BBB-permeable neuroprotectant, exerts complementary therapeutic effects by scavenging ROS and inhibiting ICAM/VCAM-mediated neutrophil adhesion^[36–38]^. Our findings demonstrated that NBP synergistically enhanced the efficacy of hUMSC-Exo by restoring AMPK phosphorylation, downregulating GSDMD and caspase-1 expression, and suppressing NLRP3 inflammasome activation.

Despite these advances, critical challenges hinder the translational potential of exosome-based therapies: (1) Naturally secreted exosomes are produced in limited quantities by cells, posing significant challenges for large-scale manufacturing. Additionally, exosome characteristics, such as size, composition, and biological activity, may vary depending on the cell source and culture conditions, leading to batch-to-batch heterogeneity. Ensuring consistent quality and functionality during scale-up remains a major hurdle for clinical translation. (2) While hUMSC-derived exosomes are generally considered safe, certain cell sources (*e*.*g*., tumor-derived exosomes) may raise potential safety risks. Furthermore, allogeneic exosomes may elicit immune responses upon repeated administration, potentially limiting their therapeutic utility^[39–40]^. Chemical modifications or excessive cargo loading may also compromise biocompatibility, necessitating rigorous preclinical evaluation^[41]^. Given these complexities, the regulatory landscape for exosome therapies is rapidly evolving to balance innovation with patient safety. Regulatory agencies emphasize the need for standardized manufacturing protocols, robust characterization, and comprehensive safety assessments. Proactive collaboration among researchers, industry stakeholders, and regulators will be essential to advance exosome-based therapies from bench to bedside.

**Figure 6:**
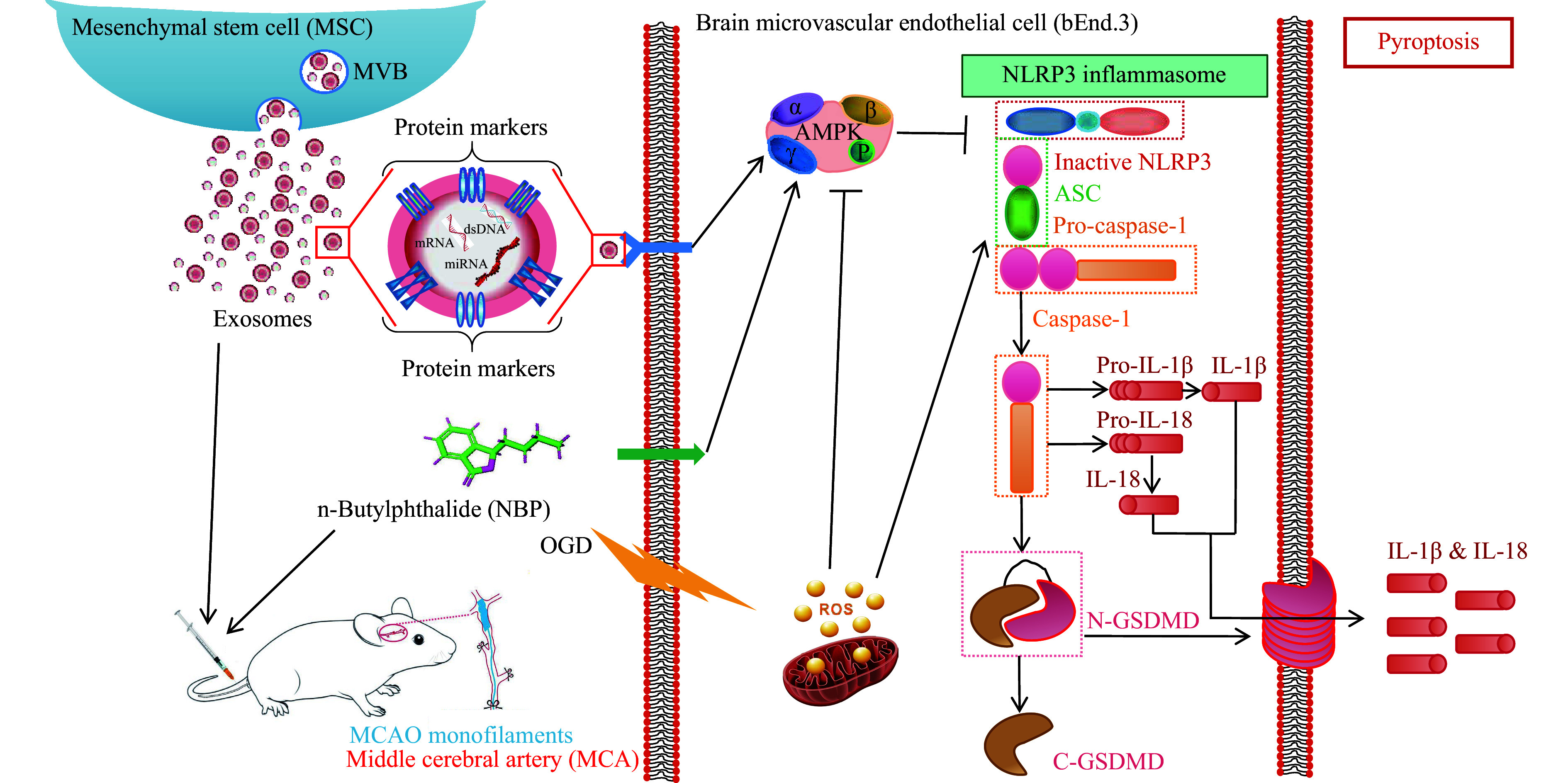
Schematic diagram of the oxygen-glucose deprivation (OGD) and post-ischemic exosome-mediated AMPK/NLRP3-associated pyroptosis pathway. hUMSC-Exos activate AMPK phosphorylation and inhibit NLRP3 inflammasome activation, thereby reducing the cleavage of caspase-1 and GSDMD, inhibiting inflammatory factor release and pyroptosis, and ultimately exerting a protective effect on cerebral ischemic injury. AMPK expression is significantly increased in the early stages of OGD exposure in both bEnd.3 cells and ischemic stroke mice. Elevated AMPK levels stimulate ROS generation, exacerbating oxidative stress and promoting NLRP3 inflammasome activation. This subsequently triggers the activation of pro-caspase-1 to its mature form. The activated caspase-1 facilitates the cleavage of pro-IL-1β while simultaneously inducing GSDMD proteolysis to release N-terminal GSDMD fragments. These processes collectively amplify the release of inflammatory factors, ultimately leading to pyroptotic cell death.

The cerebral microvascular endothelium serves as both a target and a propagator of post-ischemic inflammation. NLRP3 inflammasome activation in endothelial cells drives BBB disruption *via* caspase-1-dependent GSDMD pore formation, facilitating leukocyte infiltration and cytokine storms^[42–43]^. Our *in* *vitro* and *in* *vivo* models demonstrated that hUMSC-Exo/NBP co-treatment significantly downregulated NLRP3, GSDMD-N, and cleaved caspase-1 expression, while significantly suppressing IL-1β (*P* < 0.05) and LDH release (*P* < 0.01). These effects mirror findings in *NLRP3* knockout models, where attenuated pyroptosis correlates with improved neurovascular outcomes^[44–45]^. Mechanistically, AMPK dysfunction serves as a critical regulator of NLRP3 hyperactivation in post-ischemic injury. As a metabolic sensor, AMPK regulates NLRP3 priming through ROS suppression and mitochondrial stabilization^[46]^. In the present study, OGD and tMCAO induced AMPK dephosphorylation, triggering ROS/ATP accumulation and NLRP3 inflammasome assembly. hUMSC-Exo/NBP co-treatment reversed this cascade, restoring p-AMPK levels and mitochondrial integrity. This aligns with findings that AMPK activators (*e*.*g*., metformin) mitigate IRI by blocking NLRP3 oligomerization^[47]^, while AMPK inhibitors exacerbate pyroptosis in chronic inflammatory models^[48]^. As depicted in ***Fig. 6***, OGD and post-ischemic exosome stimulation activated AMPK in the early phase, which subsequently induced ROS generation and NLRP3 inflammasome activation, leading to caspase-1 maturation, GSDMD cleavage, and pyroptotic cell death.

In conclusion, the present study suggests that hUMSC-Exo/NBP co-treatment exerts neuroprotective and anti-inflammatory effects against post-ischemic injury by inhibiting AMPK-mediated NLRP3 inflammasome activation. These insights may enhance our pharmacological understanding of NBP and exosomes, laying a foundation for novel combinatorial therapies to improve ischemic stroke outcomes.

## Additional information

The online version contains supplementary materials available at http://www.jbr-pub.org.cn/article/doi/10.7555/JBR.39.20250189?pageType=en.
