# Supplementary material for: hUMSC-derived exosomes alleviate hypoxic cerebrovascular injury via AMPK/NLRP3-mediated pyroptosis suppression and mitochondrial protection
Source: J Biomed Res. 2026 May 21;40(3):312–26. doi: 10.7555/JBR.39.20250189 (PMC13231368; doi:10.7555/JBR.39.20250189)
Supplement: Supplementary file 1 — The online version contains supplementary materials available at http://www.jbr-pub.org.cn/article/doi/10.7555/JBR.39.20250189?pageType=en. [file jbr-40-3-312-S1.pdf]

Available online at www.jbr-pub.org.cn

Open Access at PubMed Central

Journal of Biomedical Research, 2026 40(3): 1–2

Supplementary Data

JBR

hUMSC-derived exosomes alleviate hypoxic cerebrovascular injury *via* AMPK/NLRP3-mediated pyroptosis suppression and mitochondrial protection

Jinteng Liu[1], Yunlong Pan[2], Haolin Wu[3], Qingyun Guo[4], Xingyue Fang[1], Yingmei Lu[2,✉], Qibing Liu[1,✉]

[1]*Department of Pharmacy & Engineering Research Center of Tropical Medicine Innovation and Transformation, the First Affiliated Hospital & School of Pharmacy of Hainan Medical University, Haikou, Hainan 570102, China;*
[2]*Key Laboratory of Modern Toxicology of Ministry of Education; School of Basic Medical Sciences, Nanjing Medical University, Nanjing, Jiangsu 211166, China;*
[3]*International Research Center on Aging and Tumor, Hainan Medical University, Haikou, Hainan 571199, China;*
[4]*Key Laboratory of Brain Science Research & Transformation in Tropical Environment of Hainan Province, Hainan Medical University, Haikou, Hainan 571199, China.*

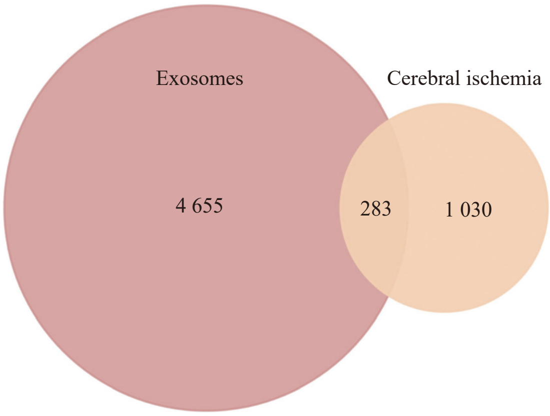

Supplementary Fig. 1 **Analysis of cerebral ischemia-associated genes.** A total of 4 655 exosome-related genes and 1 313 cerebral ischemia-related genes were identified by mining the DisGeNET database. Venn diagram analysis revealed 283 overlapping genes.

✉Corresponding authors: Yingmei Lu, E-mail: lufx@njmu.edu.cn, ORCID: 0000-0003-3187-4344; Qibing Liu, E-mail: qibing.liu@hainmc.edu.cn, ORCID: 0000-0003-4965-0007.

Received: 01 May 2025; Revised: 24 August 2025; Accepted: 25 August 2025; Available online: 28 August 2025; Published date: 21 May 2026

CLC number: R743.3, Document code: A

The authors reported no conflict of interests.

This is an open access article under the Creative Commons Attribution (CC BY 4.0) license, which permits others to distribute, remix, adapt and build upon this work, for commercial use, provided the original work is properly cited.

© 2026 by Journal of Biomedical Research.

https://doi.org/10.7555/JBR.39.20250189

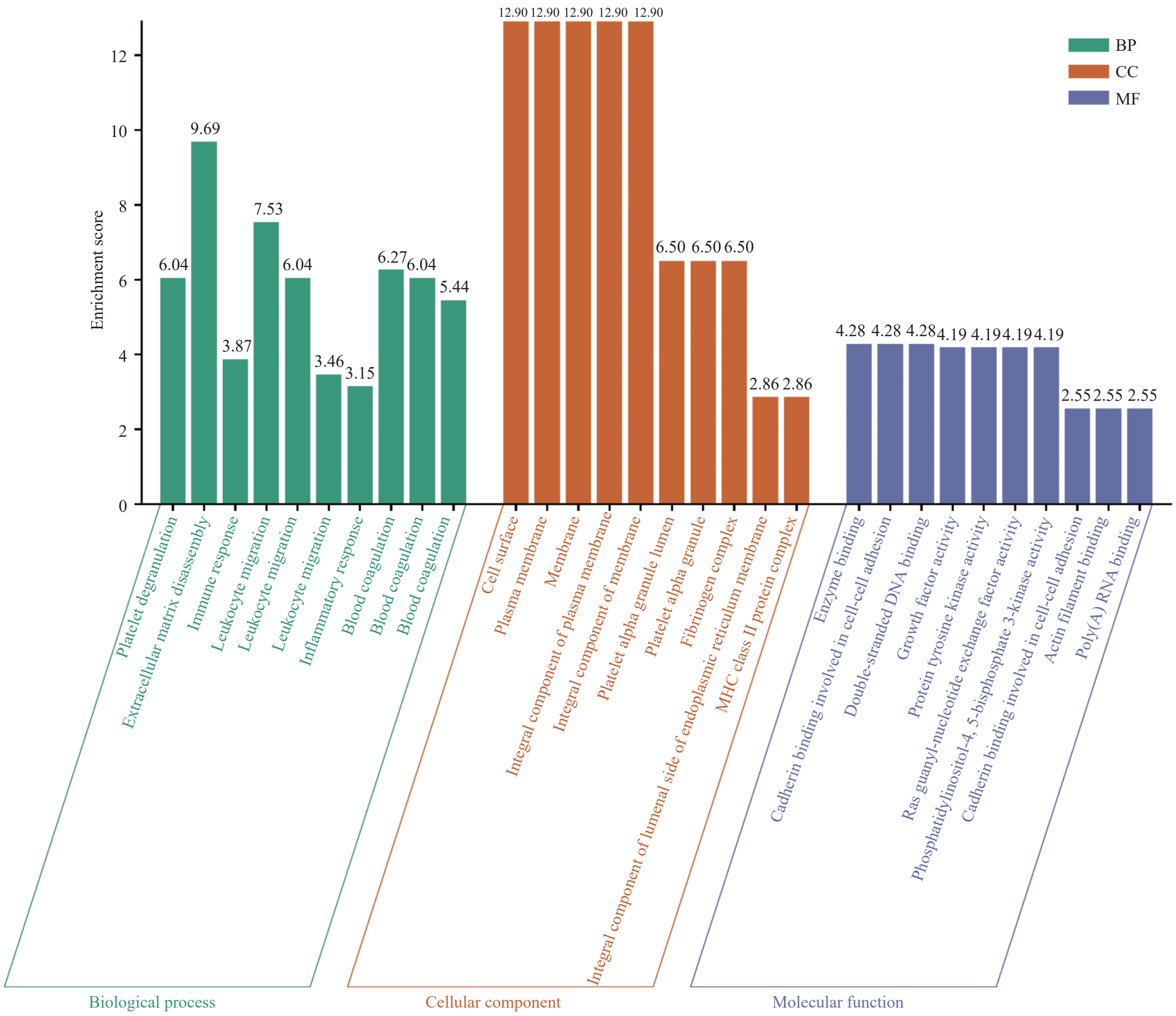

Supplementary Fig. 2 **Gene Ontology (GO) enrichment analysis.** Exosome-associated biological processes (BP) were predominantly linked to cerebral ischemia, with immune and inflammatory responses dominated. At the cellular component (CC) level, significant enrichment was observed in plasma membrane-related structures, while molecular functions (MF) were primarily associated with protein-protein interactions.
